# Tetra­kis(μ-3,4-dimeth­oxy­phenyl­acetato)­bis­[(3,4-dimeth­oxy­phenyl­acetato)(1,10-phenanthroline)holmium(III)]

**DOI:** 10.1107/S1600536810036408

**Published:** 2010-09-18

**Authors:** Guo-Liang Zhao, Jia-Lu Liu, Jian-Feng Liu

**Affiliations:** a College of Chemistry and Life Sciences, Zhejiang Normal University and Zhejiang Normal University Xingzhi College, Jinhua, Zhejiang 321004, People’s Republic of China

## Abstract

In the centrosymmetric title compound, [Ho_2_(C_10_H_11_O_4_)_6_(C_12_H_8_N_2_)_2_], the Ho^III^ atom is nine-coordinated by seven O atoms from the 3,4-dimeth­oxy­phenyl­acetate (*L*) anions and two N atoms from a 1,10-phenanthroline (phen) mol­ecule. The *L* ligands are coordinated to the Ho^III^ ions in three modes: chelating, bridging and bridging–tridentate. Intra­molecular C—H⋯O inter­actions occur. The crystal packing is stabilized by inter­molecular C—H⋯O inter­actions and weak aromatic π–π inter­actions between phen mol­ecules and the aromatic rings of the *L* ligands [centroid–centroid distance = 3.821 (2) Å].

## Related literature

For related structures, see: Li *et al.* (2005 ▶); Li & Zou (2005 ▶); Wang *et al.* (2010 ▶); Liu *et al.* (2010 ▶).
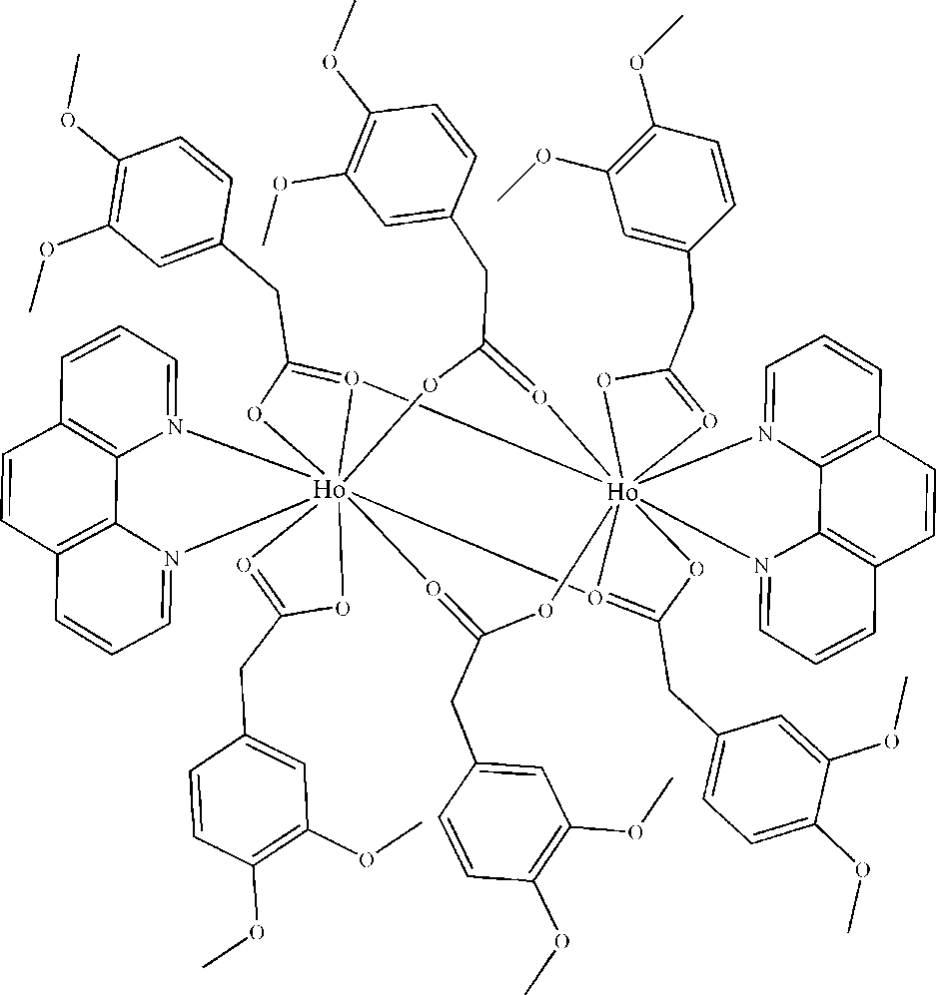


## Experimental

### 

#### Crystal data

[Ho_2_(C_10_H_11_O_4_)_6_(C_12_H_8_N_2_)_2_]
*M* *_r_* = 1861.40
Triclinic, 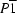

*a* = 12.3069 (2) Å
*b* = 12.3789 (2) Å
*c* = 14.6591 (2) Å
α = 91.020 (1)°
β = 103.547 (1)°
γ = 115.477 (1)°
*V* = 1942.03 (5) Å^3^
*Z* = 1
Mo *K*α radiation
μ = 2.11 mm^−1^
*T* = 296 K
0.30 × 0.16 × 0.05 mm

#### Data collection

Bruker APEXII CCD area-detector diffractometer
Absorption correction: multi-scan (*SADABS*; Sheldrick, 1996 ▶) *T*_min_ = 0.676, *T*_max_ = 0.909
31540 measured reflections
8930 independent reflections
7217 reflections with *I* > 2σ(*I*)
*R*_int_ = 0.048

#### Refinement

*R*[*F* ^2^ > 2σ(*F* ^2^)] = 0.033
*wR*(*F* ^2^) = 0.068
*S* = 1.04
8930 reflections
514 parameters
H-atom parameters constrained
Δρ_max_ = 0.92 e Å^−3^
Δρ_min_ = −0.57 e Å^−3^

### 

Data collection: *APEX2* (Bruker, 2006 ▶); cell refinement: *SAINT* (Bruker, 2006 ▶); data reduction: *SAINT*; program(s) used to solve structure: *SHELXS97* (Sheldrick, 2008 ▶); program(s) used to refine structure: *SHELXL97* (Sheldrick, 2008 ▶); molecular graphics: *SHELXTL* (Sheldrick, 2008 ▶); software used to prepare material for publication: *SHELXL97*.

## Supplementary Material

Crystal structure: contains datablocks I, global. DOI: 10.1107/S1600536810036408/pv2299sup1.cif

Structure factors: contains datablocks I. DOI: 10.1107/S1600536810036408/pv2299Isup2.hkl

Additional supplementary materials:  crystallographic information; 3D view; checkCIF report

## Figures and Tables

**Table 1:** Hydrogen-bond geometry (Å, °)

*D*—H⋯*A*	*D*—H	H⋯*A*	*D*⋯*A*	*D*—H⋯*A*
C40—H40*A*⋯O3	0.93	2.52	2.972 (4)	110
C8—H8*A*⋯O6^i^	0.96	2.55	3.319 (5)	138
C16—H16*A*⋯O4^ii^	0.93	2.51	3.410 (4)	162
C18—H18*C*⋯O4^ii^	0.96	2.36	3.266 (4)	156
C21—H21*C*⋯O1	0.96	2.83	3.291 (6)	111
C21—H21*C*⋯O2	0.96	2.82	3.749 (6)	162
C31—H31*A*⋯O11^iii^	0.93	2.37	3.008 (4)	126
C38—H38*A*⋯O7^iv^	0.93	2.36	3.215 (4)	153

Symmetry codes: (i) 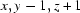; (ii) 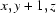; (iii) 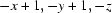; (iv) 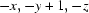.

**Fig. 1.**
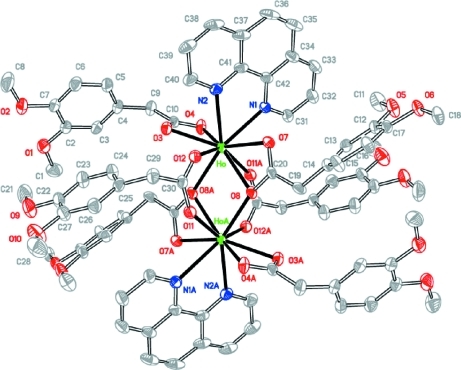
The molecular structure of the title complex. Displacement ellipsoids are drawn at the 30% probability level and H-atoms have been excluded for clarity. Symmetry code for atoms with labels ending in A: -x+1, -y+1, -z.

**Fig. 2.**
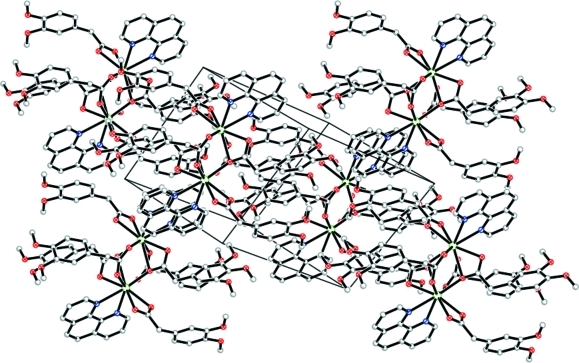
The packing plot of the title complex.

## supplementary crystallographic information

### Comment

The rare earth complexes with aromatic carboxylates have a variety of structures due to the various coordination modes of carboxylate groups, which have received considerable attention for many years (Li & Zou, 2005; Li *et al.*, 2005). We have reported some similar mixed-ligands lanthanide carboxylate complexes with dimeric structures (Wang *et al.*, 2010; Liu *et al.*, 2010). We have now prepared a new holmium complex [Ho(*L*)_3_phen]_2_, wherein *L* = 3,4-dimethoxyphenylacetate and phen = 1,10-phenanthroline]. In this paper, the crystal structure of the title complex is reported.

The structure of the title complex is shown in Fig. 1. It is a centrosymmetric dimer which consists of six 3,4-dimethoxyphenylacetate anions, two 1,10-phenanthroline molecules and two Ho^III^ ions. The Ho^III^ ion is nine-coordinated by two N atoms from one 1,10-phenanthroline and seven O atoms from carboxylate groups with a mean Ho—O bond length of 2.400 (2) Å. The ligands (*L*) are coordinated to the Ho^III^ ions in three different modes: chelating, bridging and bridging tridentate. Around each Ho^III^, there is one *L* ligand in chelating mode through two O atoms from the carboxyl group. Two symmetric *L* ligands bridge the two Ho centers though carboxyl O atoms. Two *L* ligands in bidentate mode form bonds with Ho^III^ ion with two carboxyl O atoms and simultaneously bond to another Ho^III^ ion with one of the carboxyl O atom. The Ho—Ho separation is 3.8741 (3) Å. The packing plot of the title complex is shown in Fig. 2. The most significant forces contribulting to the formation and stabilization of the crystal packing are intermolecular interactions of the type C—H···O hydrogen bonds and weak π–π aromatic interactions from phen molecules and aromatic rings of the *L* ligands. The ring [N(2)/C(40)—C(41)] stacks with its symmetry related ring of an adjacent molecule. The distance of Cg and Cg* [* = -x, 1-y, -z] being 3.821 (2) Å, where Cg is the center of the ring.

### Experimental

A mixture of 3,4-dimethoxyphenylacetic acid (0.5886 g, 3 mmol), Ho_2_O_3_ (0.1889 g, 0.5 mmol), 1,10-phenanthroline (0.1982 g, 1 mmol) and purified water (20 ml) was sealed in a 25 ml stainless steel reactor and kept at 433 K for 3 d. The reactor was cooled to room temperature at a speed of 5 ° per hour. A few colourless single crystals were obtained from the solution.

### Refinement

The H atoms were positioned geometrically and refined using a riding model with C—H distances 0.93, 0.96 and 0.97 Å for aryl, methyl and methylene τype H-atoms, respectvely, and  *U*_iso_(H) = 1.5*U*_eq_(methyl C) or 1.2*U*_eq_(methylene and aryl C)

### Crystal data

**Table:**

[Ho_2_(C_10_H_11_O_4_)_6_(C_12_H_8_N_2_)_2_]	*Z* = 1
*M**_r_* = 1861.40	*F*(000) = 940
Triclinic, *P*1	*D*_x_ = 1.592 Mg m^−^^3^
Hall symbol: -P 1	Mo *K*α radiation, λ = 0.71073 Å
*a* = 12.3069 (2) Å	Cell parameters from 6803 reflections
*b* = 12.3789 (2) Å	θ = 1.4–27.6°
*c* = 14.6591 (2) Å	µ = 2.11 mm^−^^1^
α = 91.020 (1)°	*T* = 296 K
β = 103.547 (1)°	Block, colourless
γ = 115.477 (1)°	0.30 × 0.16 × 0.05 mm
*V* = 1942.03 (5) Å^3^	

### Data collection

**Table:**

Bruker APEXII CCD area-detector diffractometer	8930 independent reflections
Radiation source: fine-focus sealed tube	7217 reflections with *I* > 2σ(*I*)
graphite	*R*_int_ = 0.048
φ and ω scans	θ_max_ = 27.6°, θ_min_ = 1.4°
Absorption correction: multi-scan (*SADABS*; Sheldrick, 1996)	*h* = −16→15
*T*_min_ = 0.676, *T*_max_ = 0.909	*k* = −16→16
31540 measured reflections	*l* = −18→19

### Refinement

**Table:**

Refinement on *F*^2^	Primary atom site location: structure-invariant direct methods
Least-squares matrix: full	Secondary atom site location: difference Fourier map
*R*[*F*^2^ > 2σ(*F*^2^)] = 0.033	Hydrogen site location: inferred from neighbouring sites
*wR*(*F*^2^) = 0.068	H-atom parameters constrained
*S* = 1.04	*w* = 1/[σ^2^(*F*_o_^2^) + (0.0272*P*)^2^] where *P* = (*F*_o_^2^ + 2*F*_c_^2^)/3
8930 reflections	(Δ/σ)_max_ = 0.001
514 parameters	Δρ_max_ = 0.92 e Å^−^^3^
0 restraints	Δρ_min_ = −0.57 e Å^−^^3^

### Special details

**Table:**

Geometry. All e.s.d.'s (except the e.s.d. in the dihedral angle between two l.s. planes) are estimated using the full covariance matrix. The cell e.s.d.'s are taken into account individually in the estimation of e.s.d.'s in distances, angles and torsion angles; correlations between e.s.d.'s in cell parameters are only used when they are defined by crystal symmetry. An approximate (isotropic) treatment of cell e.s.d.'s is used for estimating e.s.d.'s involving l.s. planes.
Refinement. Refinement of *F*^2^ against ALL reflections. The weighted *R*-factor *wR* and goodness of fit *S* are based on *F*^2^, conventional *R*-factors *R* are based on *F*, with *F* set to zero for negative *F*^2^. The threshold expression of *F*^2^ > σ(*F*^2^) is used only for calculating *R*-factors(gt) *etc*. and is not relevant to the choice of reflections for refinement. *R*-factors based on *F*^2^ are statistically about twice as large as those based on *F*, and *R*- factors based on ALL data will be even larger.

### Fractional atomic coordinates and isotropic or equivalent isotropic displacement parameters (Å^2^)

**Table:**

	*x*	*y*	*z*	*U*_iso_*/*U*_eq_	
Ho	0.322299 (13)	0.394135 (14)	−0.031917 (10)	0.03732 (6)	
N1	0.1393 (2)	0.3395 (2)	−0.18380 (19)	0.0423 (6)	
O1	0.3971 (2)	−0.0570 (3)	0.33827 (19)	0.0714 (8)	
C1	0.4836 (4)	−0.0673 (4)	0.2938 (3)	0.0801 (13)	
H1A	0.5372	−0.0928	0.3367	0.120*	
H1B	0.5331	0.0096	0.2766	0.120*	
H1C	0.4391	−0.1257	0.2379	0.120*	
N2	0.1144 (2)	0.3556 (2)	−0.0045 (2)	0.0460 (7)	
O2	0.2650 (3)	−0.0150 (3)	0.43207 (18)	0.0762 (8)	
C2	0.3127 (3)	−0.0213 (3)	0.2872 (3)	0.0519 (9)	
O3	0.2761 (2)	0.2511 (2)	0.08389 (15)	0.0446 (5)	
C3	0.2930 (3)	−0.0101 (3)	0.1925 (2)	0.0506 (8)	
H3A	0.3412	−0.0257	0.1587	0.061*	
O4	0.2375 (2)	0.1805 (2)	−0.06397 (15)	0.0523 (6)	
C4	0.2034 (3)	0.0236 (3)	0.1462 (2)	0.0482 (8)	
O5	0.1107 (2)	0.7367 (2)	−0.36823 (16)	0.0632 (7)	
C5	0.1341 (3)	0.0471 (3)	0.1985 (3)	0.0586 (10)	
H5A	0.0735	0.0702	0.1686	0.070*	
O6	0.1314 (2)	0.9430 (2)	−0.30608 (15)	0.0489 (6)	
C6	0.1524 (4)	0.0372 (3)	0.2934 (3)	0.0594 (10)	
H6A	0.1052	0.0544	0.3271	0.071*	
O7	0.29365 (19)	0.57028 (19)	−0.08541 (15)	0.0450 (5)	
C7	0.2409 (3)	0.0017 (3)	0.3386 (2)	0.0521 (9)	
O8	0.48677 (19)	0.59853 (19)	−0.04397 (14)	0.0429 (5)	
C8	0.1892 (5)	−0.0028 (5)	0.4850 (3)	0.0941 (15)	
H8A	0.2158	−0.0167	0.5488	0.141*	
H8B	0.1040	−0.0606	0.4572	0.141*	
H8C	0.1956	0.0773	0.4854	0.141*	
O9	0.6574 (3)	0.3196 (3)	0.50641 (19)	0.0787 (8)	
C9	0.1854 (4)	0.0357 (3)	0.0430 (2)	0.0548 (9)	
H9A	0.0968	−0.0057	0.0115	0.066*	
H9B	0.2257	−0.0048	0.0167	0.066*	
C10	0.2361 (3)	0.1644 (3)	0.0208 (2)	0.0411 (7)	
O10	0.7495 (3)	0.5463 (3)	0.5641 (2)	0.0917 (10)	
C11	0.0784 (4)	0.6144 (3)	−0.3965 (3)	0.0790 (14)	
H11A	0.0167	0.5865	−0.4566	0.118*	
H11B	0.0453	0.5662	−0.3501	0.118*	
H11C	0.1515	0.6075	−0.4021	0.118*	
O11	0.5943 (2)	0.6253 (2)	0.15514 (14)	0.0491 (6)	
C12	0.1977 (3)	0.7908 (3)	−0.2841 (2)	0.0404 (7)	
O12	0.3859 (2)	0.5183 (2)	0.11182 (15)	0.0440 (5)	
C13	0.2748 (3)	0.7456 (3)	−0.2339 (2)	0.0414 (7)	
H13A	0.2698	0.6732	−0.2581	0.050*	
C14	0.3601 (3)	0.8069 (3)	−0.1476 (2)	0.0421 (7)	
C15	0.3611 (3)	0.9104 (3)	−0.1123 (2)	0.0547 (9)	
H15A	0.4136	0.9494	−0.0528	0.066*	
C16	0.2862 (3)	0.9592 (3)	−0.1626 (2)	0.0495 (9)	
H16A	0.2905	1.0309	−0.1375	0.059*	
C17	0.2062 (3)	0.9011 (3)	−0.2490 (2)	0.0375 (7)	
C18	0.1639 (4)	1.0674 (3)	−0.2852 (2)	0.0566 (10)	
H18A	0.1061	1.0873	−0.3290	0.085*	
H18B	0.2469	1.1155	−0.2907	0.085*	
H18C	0.1606	1.0833	−0.2218	0.085*	
C19	0.4543 (3)	0.7644 (3)	−0.0977 (3)	0.0516 (9)	
H19A	0.5109	0.7745	−0.1367	0.062*	
H19B	0.5033	0.8180	−0.0388	0.062*	
C20	0.4062 (3)	0.6372 (3)	−0.0751 (2)	0.0369 (7)	
C21	0.6063 (5)	0.1934 (4)	0.4770 (3)	0.0975 (16)	
H21A	0.6440	0.1579	0.5241	0.146*	
H21B	0.6228	0.1806	0.4179	0.146*	
H21C	0.5178	0.1567	0.4692	0.146*	
C22	0.6133 (3)	0.3835 (4)	0.4469 (2)	0.0568 (9)	
C23	0.5234 (4)	0.3343 (4)	0.3615 (3)	0.0631 (10)	
H23A	0.4896	0.2522	0.3409	0.076*	
C24	0.4832 (4)	0.4065 (3)	0.3063 (2)	0.0569 (9)	
H24A	0.4225	0.3723	0.2490	0.068*	
C25	0.5318 (3)	0.5279 (3)	0.3352 (2)	0.0463 (8)	
C26	0.6210 (3)	0.5777 (4)	0.4211 (2)	0.0560 (9)	
H26A	0.6544	0.6599	0.4415	0.067*	
C27	0.6610 (3)	0.5055 (4)	0.4772 (3)	0.0598 (10)	
C28	0.7925 (5)	0.6654 (5)	0.6041 (3)	0.0988 (17)	
H28A	0.8530	0.6815	0.6638	0.148*	
H28B	0.7236	0.6772	0.6136	0.148*	
H28C	0.8304	0.7195	0.5622	0.148*	
C29	0.4864 (3)	0.6051 (3)	0.2731 (2)	0.0514 (9)	
H29A	0.5396	0.6898	0.2974	0.062*	
H29B	0.4020	0.5868	0.2747	0.062*	
C30	0.4886 (3)	0.5814 (3)	0.1712 (2)	0.0426 (8)	
C31	0.1508 (3)	0.3323 (3)	−0.2708 (2)	0.0501 (8)	
H31A	0.2250	0.3356	−0.2788	0.060*	
C32	0.0572 (3)	0.3199 (3)	−0.3513 (3)	0.0599 (10)	
H32A	0.0678	0.3122	−0.4114	0.072*	
C33	−0.0500 (3)	0.3191 (3)	−0.3402 (3)	0.0611 (11)	
H33A	−0.1130	0.3118	−0.3930	0.073*	
C34	−0.0651 (3)	0.3292 (3)	−0.2503 (3)	0.0515 (9)	
C35	−0.1740 (3)	0.3324 (3)	−0.2336 (4)	0.0700 (12)	
H35A	−0.2363	0.3308	−0.2845	0.084*	
C36	−0.1871 (3)	0.3376 (3)	−0.1459 (4)	0.0727 (13)	
H36A	−0.2588	0.3393	−0.1370	0.087*	
C37	−0.0930 (3)	0.3408 (3)	−0.0650 (3)	0.0589 (10)	
C38	−0.1051 (4)	0.3425 (3)	0.0270 (4)	0.0698 (12)	
H38A	−0.1771	0.3404	0.0384	0.084*	
C39	−0.0106 (4)	0.3474 (3)	0.1002 (3)	0.0680 (11)	
H39A	−0.0189	0.3450	0.1616	0.082*	
C40	0.0991 (4)	0.3561 (3)	0.0812 (3)	0.0586 (10)	
H40A	0.1643	0.3625	0.1318	0.070*	
C41	0.0185 (3)	0.3453 (3)	−0.0790 (3)	0.0469 (8)	
C42	0.0318 (3)	0.3382 (3)	−0.1724 (2)	0.0427 (8)	

### Atomic displacement parameters (Å^2^)

**Table:**

	*U*^11^	*U*^22^	*U*^33^	*U*^12^	*U*^13^	*U*^23^
Ho	0.03932 (9)	0.05518 (10)	0.03659 (8)	0.03442 (8)	0.01714 (6)	0.01890 (6)
N1	0.0390 (15)	0.0474 (16)	0.0518 (16)	0.0277 (13)	0.0149 (12)	0.0178 (13)
O1	0.0688 (18)	0.093 (2)	0.0748 (18)	0.0511 (17)	0.0269 (15)	0.0375 (16)
C1	0.066 (3)	0.085 (3)	0.104 (4)	0.044 (3)	0.027 (3)	0.022 (3)
N2	0.0441 (16)	0.0546 (17)	0.0583 (17)	0.0318 (14)	0.0273 (14)	0.0200 (14)
O2	0.084 (2)	0.112 (2)	0.0462 (15)	0.0508 (18)	0.0265 (14)	0.0322 (15)
C2	0.052 (2)	0.051 (2)	0.055 (2)	0.0238 (18)	0.0177 (17)	0.0207 (17)
O3	0.0548 (14)	0.0514 (14)	0.0384 (12)	0.0304 (12)	0.0179 (10)	0.0136 (11)
C3	0.062 (2)	0.047 (2)	0.053 (2)	0.0265 (18)	0.0280 (18)	0.0170 (16)
O4	0.0747 (17)	0.0616 (15)	0.0350 (12)	0.0401 (14)	0.0204 (11)	0.0174 (11)
C4	0.060 (2)	0.0419 (19)	0.0453 (19)	0.0216 (17)	0.0204 (17)	0.0159 (15)
O5	0.0823 (18)	0.0458 (15)	0.0519 (14)	0.0359 (14)	−0.0129 (13)	−0.0013 (11)
C5	0.065 (2)	0.068 (3)	0.055 (2)	0.037 (2)	0.0204 (19)	0.0264 (19)
O6	0.0580 (14)	0.0515 (14)	0.0470 (13)	0.0383 (12)	0.0038 (11)	0.0108 (11)
C6	0.070 (3)	0.072 (3)	0.055 (2)	0.041 (2)	0.0306 (19)	0.0192 (19)
O7	0.0383 (12)	0.0530 (14)	0.0573 (14)	0.0313 (11)	0.0146 (10)	0.0205 (11)
C7	0.057 (2)	0.058 (2)	0.0423 (19)	0.0227 (19)	0.0193 (17)	0.0169 (17)
O8	0.0449 (12)	0.0639 (15)	0.0438 (12)	0.0420 (12)	0.0187 (10)	0.0231 (11)
C8	0.128 (4)	0.120 (4)	0.053 (3)	0.063 (4)	0.041 (3)	0.025 (3)
O9	0.095 (2)	0.079 (2)	0.0620 (17)	0.0491 (18)	−0.0004 (15)	0.0195 (15)
C9	0.070 (2)	0.048 (2)	0.0428 (19)	0.0224 (19)	0.0157 (17)	0.0135 (16)
C10	0.0415 (18)	0.056 (2)	0.0399 (18)	0.0323 (17)	0.0138 (14)	0.0167 (16)
O10	0.096 (2)	0.083 (2)	0.079 (2)	0.0447 (19)	−0.0164 (18)	−0.0074 (17)
C11	0.094 (3)	0.049 (2)	0.075 (3)	0.033 (2)	−0.013 (2)	−0.013 (2)
O11	0.0441 (13)	0.0771 (17)	0.0401 (12)	0.0366 (13)	0.0169 (10)	0.0145 (11)
C12	0.0457 (18)	0.0407 (18)	0.0369 (17)	0.0226 (15)	0.0077 (14)	0.0118 (14)
O12	0.0438 (13)	0.0596 (14)	0.0450 (12)	0.0332 (12)	0.0206 (11)	0.0150 (11)
C13	0.0450 (18)	0.0344 (17)	0.0501 (19)	0.0212 (15)	0.0147 (15)	0.0109 (14)
C14	0.0394 (17)	0.0439 (19)	0.0482 (19)	0.0238 (15)	0.0101 (14)	0.0148 (15)
C15	0.059 (2)	0.057 (2)	0.0455 (19)	0.0353 (19)	−0.0074 (17)	−0.0023 (17)
C16	0.060 (2)	0.053 (2)	0.0446 (19)	0.0390 (19)	0.0057 (16)	0.0017 (16)
C17	0.0413 (17)	0.0442 (18)	0.0391 (17)	0.0281 (15)	0.0140 (14)	0.0142 (14)
C18	0.076 (3)	0.056 (2)	0.056 (2)	0.048 (2)	0.0122 (19)	0.0164 (17)
C19	0.0405 (18)	0.052 (2)	0.067 (2)	0.0290 (17)	0.0053 (16)	0.0135 (18)
C20	0.0438 (19)	0.053 (2)	0.0304 (15)	0.0353 (17)	0.0119 (13)	0.0130 (14)
C21	0.123 (4)	0.079 (3)	0.091 (4)	0.050 (3)	0.015 (3)	0.041 (3)
C22	0.063 (2)	0.068 (3)	0.042 (2)	0.034 (2)	0.0111 (17)	0.0163 (18)
C23	0.078 (3)	0.058 (2)	0.050 (2)	0.032 (2)	0.0083 (19)	0.0124 (18)
C24	0.069 (2)	0.062 (2)	0.0396 (19)	0.032 (2)	0.0085 (17)	0.0106 (17)
C25	0.052 (2)	0.065 (2)	0.0370 (17)	0.0344 (19)	0.0230 (15)	0.0170 (16)
C26	0.061 (2)	0.063 (2)	0.046 (2)	0.030 (2)	0.0136 (17)	0.0060 (18)
C27	0.058 (2)	0.078 (3)	0.047 (2)	0.036 (2)	0.0063 (18)	0.0130 (19)
C28	0.094 (4)	0.113 (4)	0.069 (3)	0.042 (3)	−0.004 (3)	−0.023 (3)
C29	0.064 (2)	0.068 (2)	0.0447 (19)	0.045 (2)	0.0251 (17)	0.0146 (17)
C30	0.051 (2)	0.061 (2)	0.0394 (17)	0.0409 (18)	0.0209 (16)	0.0197 (16)
C31	0.052 (2)	0.056 (2)	0.049 (2)	0.0308 (18)	0.0127 (16)	0.0178 (17)
C32	0.062 (2)	0.065 (2)	0.051 (2)	0.033 (2)	0.0030 (18)	0.0132 (18)
C33	0.049 (2)	0.051 (2)	0.069 (3)	0.0219 (19)	−0.0097 (19)	0.0106 (19)
C34	0.0367 (18)	0.0367 (19)	0.074 (3)	0.0171 (16)	0.0015 (17)	0.0056 (17)
C35	0.037 (2)	0.055 (2)	0.111 (4)	0.0254 (19)	−0.001 (2)	0.000 (2)
C36	0.0298 (19)	0.058 (3)	0.132 (4)	0.0247 (19)	0.014 (2)	−0.002 (3)
C37	0.039 (2)	0.039 (2)	0.105 (3)	0.0183 (17)	0.028 (2)	0.004 (2)
C38	0.047 (2)	0.057 (2)	0.125 (4)	0.029 (2)	0.047 (3)	0.010 (2)
C39	0.071 (3)	0.066 (3)	0.092 (3)	0.035 (2)	0.057 (3)	0.017 (2)
C40	0.058 (2)	0.072 (3)	0.068 (2)	0.038 (2)	0.037 (2)	0.022 (2)
C41	0.0342 (17)	0.0343 (18)	0.079 (3)	0.0187 (15)	0.0201 (17)	0.0131 (17)
C42	0.0341 (17)	0.0342 (17)	0.063 (2)	0.0193 (14)	0.0112 (15)	0.0112 (15)

### Geometric parameters (Å, °)

**Table:**

Ho—O8^i^	2.3142 (18)	C12—C13	1.377 (4)
Ho—O11^i^	2.332 (2)	C12—C17	1.403 (4)
Ho—O12	2.343 (2)	O12—C30	1.255 (4)
Ho—O4	2.378 (2)	C13—C14	1.391 (4)
Ho—O3	2.456 (2)	C13—H13A	0.9300
Ho—O7	2.4670 (19)	C14—C15	1.367 (4)
Ho—O8	2.511 (2)	C14—C19	1.516 (4)
Ho—N2	2.524 (2)	C15—C16	1.391 (4)
Ho—N1	2.603 (2)	C15—H15A	0.9300
Ho—C10	2.770 (3)	C16—C17	1.366 (4)
Ho—C20	2.867 (3)	C16—H16A	0.9300
Ho—Ho^i^	3.8741 (3)	C18—H18A	0.9600
N1—C31	1.322 (4)	C18—H18B	0.9600
N1—C42	1.366 (4)	C18—H18C	0.9600
O1—C2	1.370 (4)	C19—C20	1.500 (4)
O1—C1	1.419 (5)	C19—H19A	0.9700
C1—H1A	0.9600	C19—H19B	0.9700
C1—H1B	0.9600	C21—H21A	0.9600
C1—H1C	0.9600	C21—H21B	0.9600
N2—C40	1.314 (4)	C21—H21C	0.9600
N2—C41	1.365 (4)	C22—C23	1.382 (5)
O2—C7	1.372 (4)	C22—C27	1.383 (5)
O2—C8	1.397 (5)	C23—C24	1.384 (5)
C2—C3	1.372 (5)	C23—H23A	0.9300
C2—C7	1.395 (5)	C24—C25	1.371 (5)
O3—C10	1.244 (4)	C24—H24A	0.9300
C3—C4	1.381 (4)	C25—C26	1.384 (5)
C3—H3A	0.9300	C25—C29	1.517 (4)
O4—C10	1.264 (4)	C26—C27	1.389 (5)
C4—C5	1.383 (5)	C26—H26A	0.9300
C4—C9	1.496 (4)	C28—H28A	0.9600
O5—C12	1.361 (3)	C28—H28B	0.9600
O5—C11	1.416 (4)	C28—H28C	0.9600
C5—C6	1.372 (5)	C29—C30	1.527 (4)
C5—H5A	0.9300	C29—H29A	0.9700
O6—C17	1.372 (3)	C29—H29B	0.9700
O6—C18	1.421 (4)	C31—C32	1.395 (4)
C6—C7	1.380 (5)	C31—H31A	0.9300
C6—H6A	0.9300	C32—C33	1.362 (5)
O7—C20	1.240 (4)	C32—H32A	0.9300
O8—C20	1.275 (3)	C33—C34	1.384 (5)
O8—Ho^i^	2.3142 (18)	C33—H33A	0.9300
C8—H8A	0.9600	C34—C42	1.408 (4)
C8—H8B	0.9600	C34—C35	1.433 (5)
C8—H8C	0.9600	C35—C36	1.337 (6)
O9—C22	1.361 (4)	C35—H35A	0.9300
O9—C21	1.423 (5)	C36—C37	1.437 (6)
C9—C10	1.514 (5)	C36—H36A	0.9300
C9—H9A	0.9700	C37—C38	1.391 (6)
C9—H9B	0.9700	C37—C41	1.413 (4)
O10—C27	1.385 (4)	C38—C39	1.363 (6)
O10—C28	1.396 (5)	C38—H38A	0.9300
C11—H11A	0.9600	C39—C40	1.402 (5)
C11—H11B	0.9600	C39—H39A	0.9300
C11—H11C	0.9600	C40—H40A	0.9300
O11—C30	1.258 (4)	C41—C42	1.422 (5)
O11—Ho^i^	2.332 (2)		
			
O8^i^—Ho—O11^i^	75.86 (7)	O4—C10—Ho	58.91 (17)
O8^i^—Ho—O12	75.67 (7)	C9—C10—Ho	176.4 (2)
O11^i^—Ho—O12	138.40 (8)	C27—O10—C28	118.2 (3)
O8^i^—Ho—O4	89.15 (8)	O5—C11—H11A	109.5
O11^i^—Ho—O4	79.00 (8)	O5—C11—H11B	109.5
O12—Ho—O4	129.94 (7)	H11A—C11—H11B	109.5
O8^i^—Ho—O3	75.22 (7)	O5—C11—H11C	109.5
O11^i^—Ho—O3	123.94 (7)	H11A—C11—H11C	109.5
O12—Ho—O3	76.21 (7)	H11B—C11—H11C	109.5
O4—Ho—O3	53.75 (7)	C30—O11—Ho^i^	137.0 (2)
O8^i^—Ho—O7	124.23 (7)	O5—C12—C13	125.1 (3)
O11^i^—Ho—O7	93.63 (8)	O5—C12—C17	115.0 (3)
O12—Ho—O7	78.23 (7)	C13—C12—C17	119.9 (3)
O4—Ho—O7	143.26 (7)	C30—O12—Ho	135.23 (19)
O3—Ho—O7	142.10 (7)	C12—C13—C14	120.9 (3)
O8^i^—Ho—O8	73.27 (7)	C12—C13—H13A	119.5
O11^i^—Ho—O8	71.50 (8)	C14—C13—H13A	119.5
O12—Ho—O8	71.63 (7)	C15—C14—C13	118.0 (3)
O4—Ho—O8	148.46 (7)	C15—C14—C19	121.1 (3)
O3—Ho—O8	139.35 (7)	C13—C14—C19	120.8 (3)
O7—Ho—O8	51.86 (6)	C14—C15—C16	122.1 (3)
O8^i^—Ho—N2	141.50 (8)	C14—C15—H15A	118.9
O11^i^—Ho—N2	139.60 (8)	C16—C15—H15A	118.9
O12—Ho—N2	78.71 (8)	C17—C16—C15	119.6 (3)
O4—Ho—N2	85.70 (8)	C17—C16—H16A	120.2
O3—Ho—N2	71.06 (8)	C15—C16—H16A	120.2
O7—Ho—N2	76.86 (8)	C16—C17—O6	124.6 (3)
O8—Ho—N2	124.29 (7)	C16—C17—C12	119.3 (3)
O8^i^—Ho—N1	150.25 (8)	O6—C17—C12	116.1 (3)
O11^i^—Ho—N1	75.80 (8)	O6—C18—H18A	109.5
O12—Ho—N1	133.15 (7)	O6—C18—H18B	109.5
O4—Ho—N1	76.77 (8)	H18A—C18—H18B	109.5
O3—Ho—N1	114.33 (8)	O6—C18—H18C	109.5
O7—Ho—N1	66.55 (7)	H18A—C18—H18C	109.5
O8—Ho—N1	105.76 (7)	H18B—C18—H18C	109.5
N2—Ho—N1	64.32 (9)	C20—C19—C14	118.1 (3)
O8^i^—Ho—C10	81.66 (8)	C20—C19—H19A	107.8
O11^i^—Ho—C10	102.29 (9)	C14—C19—H19A	107.8
O12—Ho—C10	102.89 (9)	C20—C19—H19B	107.8
O4—Ho—C10	27.08 (8)	C14—C19—H19B	107.8
O3—Ho—C10	26.68 (8)	H19A—C19—H19B	107.1
O7—Ho—C10	152.62 (8)	O7—C20—O8	120.0 (3)
O8—Ho—C10	154.93 (7)	O7—C20—C19	123.0 (2)
N2—Ho—C10	76.58 (8)	O8—C20—C19	117.0 (3)
N1—Ho—C10	95.73 (8)	O7—C20—Ho	58.91 (15)
O8^i^—Ho—C20	99.25 (8)	O8—C20—Ho	61.05 (16)
O11^i^—Ho—C20	81.94 (8)	C19—C20—Ho	178.0 (2)
O12—Ho—C20	73.44 (8)	O9—C21—H21A	109.5
O4—Ho—C20	156.61 (8)	O9—C21—H21B	109.5
O3—Ho—C20	149.55 (8)	H21A—C21—H21B	109.5
O7—Ho—C20	25.49 (7)	O9—C21—H21C	109.5
O8—Ho—C20	26.38 (7)	H21A—C21—H21C	109.5
N2—Ho—C20	100.42 (8)	H21B—C21—H21C	109.5
N1—Ho—C20	85.54 (8)	O9—C22—C23	124.6 (4)
C10—Ho—C20	175.76 (9)	O9—C22—C27	116.4 (3)
O8^i^—Ho—Ho^i^	38.38 (5)	C23—C22—C27	119.0 (3)
O11^i^—Ho—Ho^i^	69.41 (5)	C22—C23—C24	120.4 (4)
O12—Ho—Ho^i^	69.39 (5)	C22—C23—H23A	119.8
O4—Ho—Ho^i^	122.93 (6)	C24—C23—H23A	119.8
O3—Ho—Ho^i^	109.84 (5)	C25—C24—C23	120.8 (3)
O7—Ho—Ho^i^	86.31 (5)	C25—C24—H24A	119.6
O8—Ho—Ho^i^	34.89 (4)	C23—C24—H24A	119.6
N2—Ho—Ho^i^	146.38 (6)	C24—C25—C26	119.1 (3)
N1—Ho—Ho^i^	134.01 (6)	C24—C25—C29	119.7 (3)
C10—Ho—Ho^i^	120.04 (6)	C26—C25—C29	121.2 (3)
C20—Ho—Ho^i^	61.00 (6)	C25—C26—C27	120.3 (4)
C31—N1—C42	118.1 (3)	C25—C26—H26A	119.8
C31—N1—Ho	123.9 (2)	C27—C26—H26A	119.8
C42—N1—Ho	117.1 (2)	C22—C27—O10	114.8 (3)
C2—O1—C1	117.6 (3)	C22—C27—C26	120.3 (3)
O1—C1—H1A	109.5	O10—C27—C26	124.8 (4)
O1—C1—H1B	109.5	O10—C28—H28A	109.5
H1A—C1—H1B	109.5	O10—C28—H28B	109.5
O1—C1—H1C	109.5	H28A—C28—H28B	109.5
H1A—C1—H1C	109.5	O10—C28—H28C	109.5
H1B—C1—H1C	109.5	H28A—C28—H28C	109.5
C40—N2—C41	118.5 (3)	H28B—C28—H28C	109.5
C40—N2—Ho	121.5 (2)	C25—C29—C30	110.6 (3)
C41—N2—Ho	119.7 (2)	C25—C29—H29A	109.5
C7—O2—C8	118.0 (3)	C30—C29—H29A	109.5
O1—C2—C3	125.5 (3)	C25—C29—H29B	109.5
O1—C2—C7	114.8 (3)	C30—C29—H29B	109.5
C3—C2—C7	119.7 (3)	H29A—C29—H29B	108.1
C10—O3—Ho	90.89 (18)	O12—C30—O11	126.1 (3)
C2—C3—C4	121.5 (3)	O12—C30—C29	117.4 (3)
C2—C3—H3A	119.2	O11—C30—C29	116.4 (3)
C4—C3—H3A	119.2	N1—C31—C32	123.2 (3)
C10—O4—Ho	94.0 (2)	N1—C31—H31A	118.4
C3—C4—C5	117.9 (3)	C32—C31—H31A	118.4
C3—C4—C9	119.9 (3)	C33—C32—C31	118.9 (4)
C5—C4—C9	122.2 (3)	C33—C32—H32A	120.6
C12—O5—C11	117.2 (3)	C31—C32—H32A	120.6
C6—C5—C4	121.7 (3)	C32—C33—C34	120.0 (3)
C6—C5—H5A	119.2	C32—C33—H33A	120.0
C4—C5—H5A	119.2	C34—C33—H33A	120.0
C17—O6—C18	116.1 (2)	C33—C34—C42	118.1 (3)
C5—C6—C7	119.9 (3)	C33—C34—C35	122.8 (4)
C5—C6—H6A	120.0	C42—C34—C35	119.1 (4)
C7—C6—H6A	120.0	C36—C35—C34	121.0 (4)
C20—O7—Ho	95.61 (16)	C36—C35—H35A	119.5
O2—C7—C6	125.3 (3)	C34—C35—H35A	119.5
O2—C7—C2	115.5 (3)	C35—C36—C37	121.5 (4)
C6—C7—C2	119.3 (3)	C35—C36—H36A	119.3
C20—O8—Ho^i^	158.2 (2)	C37—C36—H36A	119.3
C20—O8—Ho	92.57 (19)	C38—C37—C41	118.2 (4)
Ho^i^—O8—Ho	106.73 (7)	C38—C37—C36	123.2 (4)
O2—C8—H8A	109.5	C41—C37—C36	118.6 (4)
O2—C8—H8B	109.5	C39—C38—C37	119.6 (3)
H8A—C8—H8B	109.5	C39—C38—H38A	120.2
O2—C8—H8C	109.5	C37—C38—H38A	120.2
H8A—C8—H8C	109.5	C38—C39—C40	118.9 (4)
H8B—C8—H8C	109.5	C38—C39—H39A	120.6
C22—O9—C21	116.7 (3)	C40—C39—H39A	120.6
C4—C9—C10	114.9 (3)	N2—C40—C39	123.3 (4)
C4—C9—H9A	108.5	N2—C40—H40A	118.4
C10—C9—H9A	108.5	C39—C40—H40A	118.4
C4—C9—H9B	108.5	N2—C41—C37	121.4 (3)
C10—C9—H9B	108.5	N2—C41—C42	118.8 (3)
H9A—C9—H9B	107.5	C37—C41—C42	119.8 (3)
O3—C10—O4	121.3 (3)	N1—C42—C34	121.7 (3)
O3—C10—C9	121.1 (3)	N1—C42—C41	118.4 (3)
O4—C10—C9	117.6 (3)	C34—C42—C41	119.9 (3)
O3—C10—Ho	62.44 (17)		
			
O8^i^—Ho—N1—C31	−25.1 (3)	O8^i^—Ho—C10—O4	−104.69 (18)
O11^i^—Ho—N1—C31	−7.1 (2)	O11^i^—Ho—C10—O4	−31.22 (19)
O12—Ho—N1—C31	138.0 (2)	O12—Ho—C10—O4	−177.82 (17)
O4—Ho—N1—C31	−88.9 (3)	O3—Ho—C10—O4	−178.3 (3)
O3—Ho—N1—C31	−128.2 (2)	O7—Ho—C10—O4	92.9 (2)
O7—Ho—N1—C31	93.4 (3)	O8—Ho—C10—O4	−103.7 (2)
O8—Ho—N1—C31	58.6 (3)	N2—Ho—C10—O4	107.31 (19)
N2—Ho—N1—C31	179.6 (3)	N1—Ho—C10—O4	45.45 (19)
C10—Ho—N1—C31	−108.3 (3)	Ho^i^—Ho—C10—O4	−104.38 (17)
C20—Ho—N1—C31	75.7 (2)	C11—O5—C12—C13	14.0 (5)
Ho^i^—Ho—N1—C31	34.5 (3)	C11—O5—C12—C17	−166.3 (3)
O8^i^—Ho—N1—C42	166.08 (19)	O8^i^—Ho—O12—C30	22.3 (3)
O11^i^—Ho—N1—C42	−175.9 (2)	O11^i^—Ho—O12—C30	−25.8 (3)
O12—Ho—N1—C42	−30.8 (3)	O4—Ho—O12—C30	98.8 (3)
O4—Ho—N1—C42	102.3 (2)	O3—Ho—O12—C30	100.3 (3)
O3—Ho—N1—C42	63.0 (2)	O7—Ho—O12—C30	−108.0 (3)
O7—Ho—N1—C42	−75.4 (2)	O8—Ho—O12—C30	−54.5 (3)
O8—Ho—N1—C42	−110.2 (2)	N2—Ho—O12—C30	173.3 (3)
N2—Ho—N1—C42	10.8 (2)	N1—Ho—O12—C30	−149.2 (3)
C10—Ho—N1—C42	82.9 (2)	C10—Ho—O12—C30	100.1 (3)
C20—Ho—N1—C42	−93.1 (2)	C20—Ho—O12—C30	−82.1 (3)
Ho^i^—Ho—N1—C42	−134.35 (19)	Ho^i^—Ho—O12—C30	−17.5 (3)
O8^i^—Ho—N2—C40	15.4 (3)	O5—C12—C13—C14	−179.2 (3)
O11^i^—Ho—N2—C40	166.0 (2)	C17—C12—C13—C14	1.2 (5)
O12—Ho—N2—C40	−33.6 (3)	C12—C13—C14—C15	2.8 (5)
O4—Ho—N2—C40	98.6 (3)	C12—C13—C14—C19	−173.3 (3)
O3—Ho—N2—C40	45.5 (3)	C13—C14—C15—C16	−4.3 (5)
O7—Ho—N2—C40	−114.0 (3)	C19—C14—C15—C16	171.8 (3)
O8—Ho—N2—C40	−91.9 (3)	C14—C15—C16—C17	1.7 (6)
N1—Ho—N2—C40	176.0 (3)	C15—C16—C17—O6	−177.6 (3)
C10—Ho—N2—C40	72.8 (3)	C15—C16—C17—C12	2.3 (5)
C20—Ho—N2—C40	−104.2 (3)	C18—O6—C17—C16	17.2 (5)
Ho^i^—Ho—N2—C40	−52.0 (3)	C18—O6—C17—C12	−162.7 (3)
O8^i^—Ho—N2—C41	−170.87 (19)	O5—C12—C17—C16	176.6 (3)
O11^i^—Ho—N2—C41	−20.2 (3)	C13—C12—C17—C16	−3.8 (5)
O12—Ho—N2—C41	140.1 (2)	O5—C12—C17—O6	−3.5 (4)
O4—Ho—N2—C41	−87.7 (2)	C13—C12—C17—O6	176.2 (3)
O3—Ho—N2—C41	−140.7 (2)	C15—C14—C19—C20	127.4 (4)
O7—Ho—N2—C41	59.8 (2)	C13—C14—C19—C20	−56.6 (5)
O8—Ho—N2—C41	81.8 (2)	Ho—O7—C20—O8	−0.6 (3)
N1—Ho—N2—C41	−10.3 (2)	Ho—O7—C20—C19	179.3 (3)
C10—Ho—N2—C41	−113.5 (2)	Ho^i^—O8—C20—O7	−151.9 (4)
C20—Ho—N2—C41	69.6 (2)	Ho—O8—C20—O7	0.6 (3)
Ho^i^—Ho—N2—C41	121.7 (2)	Ho^i^—O8—C20—C19	28.1 (7)
C1—O1—C2—C3	9.3 (5)	Ho—O8—C20—C19	−179.3 (2)
C1—O1—C2—C7	−172.3 (3)	Ho^i^—O8—C20—Ho	−152.5 (5)
O8^i^—Ho—O3—C10	−101.02 (18)	C14—C19—C20—O7	−8.4 (5)
O11^i^—Ho—O3—C10	−39.8 (2)	C14—C19—C20—O8	171.5 (3)
O12—Ho—O3—C10	−179.55 (18)	O8^i^—Ho—C20—O7	−169.38 (17)
O4—Ho—O3—C10	−0.98 (17)	O11^i^—Ho—C20—O7	116.47 (18)
O7—Ho—O3—C10	131.53 (18)	O12—Ho—C20—O7	−97.44 (18)
O8—Ho—O3—C10	−141.18 (16)	O4—Ho—C20—O7	80.8 (3)
N2—Ho—O3—C10	97.85 (18)	O3—Ho—C20—O7	−92.9 (2)
N1—Ho—O3—C10	49.00 (19)	O8—Ho—C20—O7	−179.4 (3)
C20—Ho—O3—C10	175.93 (17)	N2—Ho—C20—O7	−22.65 (19)
Ho^i^—Ho—O3—C10	−117.85 (16)	N1—Ho—C20—O7	40.21 (18)
O1—C2—C3—C4	178.4 (3)	Ho^i^—Ho—C20—O7	−172.7 (2)
C7—C2—C3—C4	0.0 (5)	O8^i^—Ho—C20—O8	10.0 (2)
O8^i^—Ho—O4—C10	73.18 (18)	O11^i^—Ho—C20—O8	−64.16 (16)
O11^i^—Ho—O4—C10	148.94 (19)	O12—Ho—C20—O8	81.94 (16)
O12—Ho—O4—C10	2.8 (2)	O4—Ho—C20—O8	−99.8 (2)
O3—Ho—O4—C10	0.96 (16)	O3—Ho—C20—O8	86.5 (2)
O7—Ho—O4—C10	−129.84 (18)	O7—Ho—C20—O8	179.4 (3)
O8—Ho—O4—C10	128.11 (18)	N2—Ho—C20—O8	156.73 (16)
N2—Ho—O4—C10	−68.64 (19)	N1—Ho—C20—O8	−140.42 (17)
N1—Ho—O4—C10	−133.25 (19)	Ho^i^—Ho—C20—O8	6.72 (14)
C20—Ho—O4—C10	−175.08 (19)	C21—O9—C22—C23	0.3 (6)
Ho^i^—Ho—O4—C10	92.49 (18)	C21—O9—C22—C27	178.4 (4)
C2—C3—C4—C5	0.6 (5)	O9—C22—C23—C24	179.1 (4)
C2—C3—C4—C9	179.6 (3)	C27—C22—C23—C24	1.1 (6)
C3—C4—C5—C6	−0.2 (5)	C22—C23—C24—C25	0.1 (6)
C9—C4—C5—C6	−179.2 (3)	C23—C24—C25—C26	−0.8 (5)
C4—C5—C6—C7	−0.7 (6)	C23—C24—C25—C29	179.8 (3)
O8^i^—Ho—O7—C20	12.7 (2)	C24—C25—C26—C27	0.3 (5)
O11^i^—Ho—O7—C20	−62.64 (18)	C29—C25—C26—C27	179.7 (3)
O12—Ho—O7—C20	76.13 (18)	O9—C22—C27—O10	1.0 (5)
O4—Ho—O7—C20	−139.07 (18)	C23—C22—C27—O10	179.2 (4)
O3—Ho—O7—C20	124.53 (18)	O9—C22—C27—C26	−179.8 (3)
O8—Ho—O7—C20	0.35 (16)	C23—C22—C27—C26	−1.6 (6)
N2—Ho—O7—C20	157.12 (19)	C28—O10—C27—C22	−173.4 (4)
N1—Ho—O7—C20	−135.5 (2)	C28—O10—C27—C26	7.4 (6)
C10—Ho—O7—C20	171.47 (19)	C25—C26—C27—C22	0.9 (6)
Ho^i^—Ho—O7—C20	6.43 (17)	C25—C26—C27—O10	−180.0 (3)
C8—O2—C7—C6	4.3 (6)	C24—C25—C29—C30	−49.1 (4)
C8—O2—C7—C2	−174.9 (4)	C26—C25—C29—C30	131.4 (3)
C5—C6—C7—O2	−177.9 (4)	Ho—O12—C30—O11	24.1 (5)
C5—C6—C7—C2	1.3 (6)	Ho—O12—C30—C29	−154.3 (2)
O1—C2—C7—O2	−0.2 (5)	Ho^i^—O11—C30—O12	−10.5 (5)
C3—C2—C7—O2	178.3 (3)	Ho^i^—O11—C30—C29	167.9 (2)
O1—C2—C7—C6	−179.5 (3)	C25—C29—C30—O12	104.4 (3)
C3—C2—C7—C6	−0.9 (5)	C25—C29—C30—O11	−74.2 (4)
O8^i^—Ho—O8—C20	−169.7 (2)	C42—N1—C31—C32	−1.9 (5)
O11^i^—Ho—O8—C20	110.00 (17)	Ho—N1—C31—C32	−170.6 (3)
O12—Ho—O8—C20	−89.62 (17)	N1—C31—C32—C33	2.3 (5)
O4—Ho—O8—C20	131.60 (18)	C31—C32—C33—C34	−0.7 (5)
O3—Ho—O8—C20	−129.07 (17)	C32—C33—C34—C42	−0.9 (5)
O7—Ho—O8—C20	−0.34 (16)	C32—C33—C34—C35	178.2 (3)
N2—Ho—O8—C20	−28.05 (19)	C33—C34—C35—C36	177.5 (4)
N1—Ho—O8—C20	41.30 (18)	C42—C34—C35—C36	−3.4 (6)
C10—Ho—O8—C20	−170.7 (2)	C34—C35—C36—C37	0.3 (6)
Ho^i^—Ho—O8—C20	−169.7 (2)	C35—C36—C37—C38	−178.0 (4)
O8^i^—Ho—O8—Ho^i^	0.0	C35—C36—C37—C41	3.6 (6)
O11^i^—Ho—O8—Ho^i^	−80.31 (8)	C41—C37—C38—C39	−0.5 (5)
O12—Ho—O8—Ho^i^	80.07 (8)	C36—C37—C38—C39	−179.0 (4)
O4—Ho—O8—Ho^i^	−58.71 (15)	C37—C38—C39—C40	2.9 (6)
O3—Ho—O8—Ho^i^	40.63 (13)	C41—N2—C40—C39	−0.5 (5)
O7—Ho—O8—Ho^i^	169.35 (12)	Ho—N2—C40—C39	173.3 (3)
N2—Ho—O8—Ho^i^	141.64 (9)	C38—C39—C40—N2	−2.4 (6)
N1—Ho—O8—Ho^i^	−149.00 (8)	C40—N2—C41—C37	3.0 (5)
C10—Ho—O8—Ho^i^	−1.0 (2)	Ho—N2—C41—C37	−171.0 (2)
C20—Ho—O8—Ho^i^	169.7 (2)	C40—N2—C41—C42	−176.7 (3)
C3—C4—C9—C10	−105.6 (4)	Ho—N2—C41—C42	9.4 (4)
C5—C4—C9—C10	73.4 (4)	C38—C37—C41—N2	−2.4 (5)
Ho—O3—C10—O4	1.7 (3)	C36—C37—C41—N2	176.1 (3)
Ho—O3—C10—C9	−179.1 (3)	C38—C37—C41—C42	177.2 (3)
Ho—O4—C10—O3	−1.8 (3)	C36—C37—C41—C42	−4.3 (5)
Ho—O4—C10—C9	179.0 (2)	C31—N1—C42—C34	0.1 (5)
C4—C9—C10—O3	−7.7 (5)	Ho—N1—C42—C34	169.6 (2)
C4—C9—C10—O4	171.5 (3)	C31—N1—C42—C41	179.7 (3)
O8^i^—Ho—C10—O3	73.58 (17)	Ho—N1—C42—C41	−10.9 (4)
O11^i^—Ho—C10—O3	147.05 (16)	C33—C34—C42—N1	1.3 (5)
O12—Ho—C10—O3	0.45 (18)	C35—C34—C42—N1	−177.9 (3)
O4—Ho—C10—O3	178.3 (3)	C33—C34—C42—C41	−178.3 (3)
O7—Ho—C10—O3	−88.8 (2)	C35—C34—C42—C41	2.6 (5)
O8—Ho—C10—O3	74.6 (3)	N2—C41—C42—N1	1.3 (4)
N2—Ho—C10—O3	−74.42 (18)	C37—C41—C42—N1	−178.3 (3)
N1—Ho—C10—O3	−136.28 (17)	N2—C41—C42—C34	−179.1 (3)
Ho^i^—Ho—C10—O3	73.89 (18)	C37—C41—C42—C34	1.3 (5)

Symmetry codes: (i) −*x*+1, −*y*+1, −*z*.

### Hydrogen-bond geometry (Å, °)

**Table:**

*D*—H···*A*	*D*—H	H···*A*	*D*···*A*	*D*—H···*A*
C40—H40A···O3	0.93	2.52	2.972 (4)	110
C8—H8A···O6^ii^	0.96	2.55	3.319 (5)	138
C16—H16A···O4^iii^	0.93	2.51	3.410 (4)	162
C18—H18C···O4^iii^	0.96	2.36	3.266 (4)	156
C21—H21C···O1	0.96	2.83	3.291 (6)	111
C21—H21C···O2	0.96	2.82	3.749 (6)	162
C31—H31A···O11^i^	0.93	2.37	3.008 (4)	126
C38—H38A···O7^iv^	0.93	2.36	3.215 (4)	153

Symmetry codes: (ii) *x*, *y*−1, *z*+1; (iii) *x*, *y*+1, *z*; (i) −*x*+1, −*y*+1, −*z*; (iv) −*x*, −*y*+1, −*z*.
